# Cryptococcosis in patients with myasthenia gravis: clinical characteristics and management strategy

**DOI:** 10.3389/fcimb.2025.1653458

**Published:** 2025-10-30

**Authors:** Ce Zhang, Mengyao Lv, Xiaotong Zhang, Shu Wang, Chengshuai Yang, Qiuting Wang, Luyuan Ma, Ziyue Li, Caiyan Zhao, Qian Zhao, Chuan Shen

**Affiliations:** ^1^ Department of Infectious Disease, Hebei Medical University Third Hospital, Shijiazhuang, China; ^2^ Clinical Research Center for Infectious Disease of Hebei Province, Shijiazhuang, China; ^3^ Hebei Key Laboratory for Diagnosis, Treatment, Emergency Prevention and Control of Critical Infectious Diseases, Shijiazhuang, China; ^4^ Office of Quality Management and Control in Healthcare, Hebei Medical University Third Hospital, Shijiazhuang, China

**Keywords:** cryptococcosis, thymoma, myasthenia gravis, immunocompromised, antifungal

## Abstract

**Background:**

Cryptococcosis, while well-documented in immunocompromised hosts, remains a rare complication in myasthenia gravis (MG) patients undergoing immunosuppressive therapy.

**Methods:**

We reported three cases of cryptococcal infection in MG patients diagnosed via cryptococcal antigen (CrAg) testing and/or histopathology, coupled with a comprehensive literature review of 14 additional cases that highlights the diagnostic and therapeutic challenges in this population. We also explored potential immunodeficiency by whole-exome sequencing (WES).

**Results:**

The comibined cohort (median age 57.1 years) demonstrated predominant central nervous system (52.9%), pulmonary (47.1%), and cutaneous (23.5%) involvement, with disseminated disease correlating with markedly decreased CD4+ T cells counts. Diagnostic complexity arose from imaging findings mimicking malignancies. A heterozygous FAS mutation (p.S19L) was identified by WES in one of our patients; however, its association with cryptococcal infection remains unclear. Management required tailored antifungal regimens (amphotericin B, fluconazole, flucytosine) and careful therapeutic drug monitoring to address immunosuppressant interactions. Four patients received surgical management targeting the local lesions. Most cases achieved clinical resolution.

**Conclusion:**

The management of cryptococcal infection in patients with MG poses significant challenges in the context of underlying immune dysfunction and the use of immunosuppressive therapy. Within this complex clinical scenario, early recognition, multidisciplinary care, and individualized treatment strategies are paramount. They underscore the need for heightened clinical vigilance and further research to optimize outcomes in this vulnerable patient population.

## 1 Introduction

Cryptococcosis is an opportunistic fungal infection caused by *Cryptococcus* species, contributing to approximately 180,000 deaths annually worldwide (Dao et al., 2024). The World Health Organization has designated *Cryptococcus* as a critical priority pathogen. Among *Cryptococcus* species, only *Cryptococcus neoformans* (*C. neoformans*) and *Cryptococcus gattii* (*C. gattii*) are recognized as human pathogens, with *C. neoformans* causing approximately 95% of infections and *C. gattii* accounting for the remaining cases. The epidemiology of cryptococcosis demonstrates distinct patterns based on host immune status. *C. neoformans* predominantly affects immunocompromised individuals. Beyond HIV-infected patients and those with malignancies, recipients of systemic immunosuppressive therapy–including solid organ transplant recipients and patients with autoimmune disorders–constitute a significant high-risk population (Ding et al., 2017; Chang et al., 2024). Studies confirm that glucocorticoids, tumor necrosis factor-α (TNF-α) inhibitors, and calcineurin inhibitors substantially impair Th1-type immune responses and macrophage-mediated clearance, thereby significantly elevating the risk of cryptococcal infection (Meya and Williamson, 2024).

The pathogenic pathway begins with inhalation of fungal particles through the respiratory tract, leading to primary pulmonary infection. The organism's remarkable ability to survive within phagocytic cells is mediated by key virulence factors such as capsular polysaccharides, phospholipases, and ureases. Through sophisticated mechanisms including the "Trojan horse" strategy, *Cryptococcus* can disseminate from the lungs to various organ systems. The central nervous system (CNS) is the most commonly affected extrapulmonary site, though the infection may also involve the skin, lymph nodes, and bone tissue (Denham and Brown, 2018). Clinical presentation shows considerable variation depending on host immune status, with immunocompromised patients being particularly vulnerable to disseminated disease. Immunosuppressive therapy exacerbates this vulnerability. For example, glucocorticoids directly impair the secretion of interferon-γ (IFN-γ) by alveolar macrophages, thereby hindering the host's ability to eliminate cryptococcal pathogens *via* phagocytic clearance mechanisms (Rohatgi and Pirofski, 2015).

Myasthenia gravis (MG) is an autoimmune disease mediated by anti-acetylcholine receptor antibodies, which impair neuromuscular transmission and cause fatigable weakness. Although immunosuppressive therapies (e.g., glucocorticoids and immunomodulators) routinely used in MG management typically increase susceptibility to several opportunistic infections (Zhou et al., 2021; García Estévez and Pardo Fernández, 2023), cryptococcosis in this patient population remains remarkably rare in the literature.

In this study, we report case series of three patients of cryptococcosis in MG receiving immunosuppressive therapy and provide a comprehensive review of the currently published relevant literature. Their clinical characteristics and management approaches are discussed.

## 2 Materials and methods

### 2.1 Medical data extraction

We retrospectively analyzed patients with MG who were diagnosed with cryptococcosis at the Hebei Medical University Third Hospital between January 2018 and June 2025. Demographic and clinical data were extracted from the hospital’s electronic medical records. Additionally, we collected prognosis results. Cryptococcosis was diagnosed through either positive fungal culture, histopathological identification, molecular testing or cryptococcal capsular antigen (CrAg) testing, with supporting clinical presentation (e.g., fever, headache, cough, chest pain, and cutaneous nodules, etc.) and/or imaging characteristics. The Ethics Committee of Hebei Medical University Third Hospital waived the need for ethics approval for the collection, analysis and publication of the retrospectively obtained and anonymized data for non-interventional case reports. However, written informed consent was obtained from the patients or their families for publication.

### 2.2 Review of the literature

A systematic search of PubMed and Web of Science was performed for articles published in English from database inception to June 2025 using the terms "*cryptococcus*," "cryptococcosis," and "myasthenia gravis," supplemented by a manual Google Scholar search, to identify previously published cases of cryptococcal infection in MG patients.

### 2.3 Whole-exome sequencing and variant analysis

Peripheral blood was collected from two MG patients, and genomic DNA was extracted using the QIAamp whole-blood DNA extraction kit (Qiagen, GmbH, Hilden, Germany). A library with an insert size of 200 bp was constructed, and target sequences were captured using high-performance liquid-phase probes. The library was then subjected to paired-end (PE) sequencing (2×150 bp) on the Illumina HiSeq X next-generation sequencing platform. The sequencing data in this study were analyzed based on the GRCh38.p13 reference genome. By integrating multiple public databases (dbSNP, COSMIC, ClinVar) and Shanghai Dishuo Becken Biotechnology Company's proprietary database, the data were processed, annotated, and subjected to bioinformatics analysis to generate objective and reliable analytical results.

## 3 Results

### 3.1 Case presentations

#### 3.1.1 Case 1

**Figure 1:**
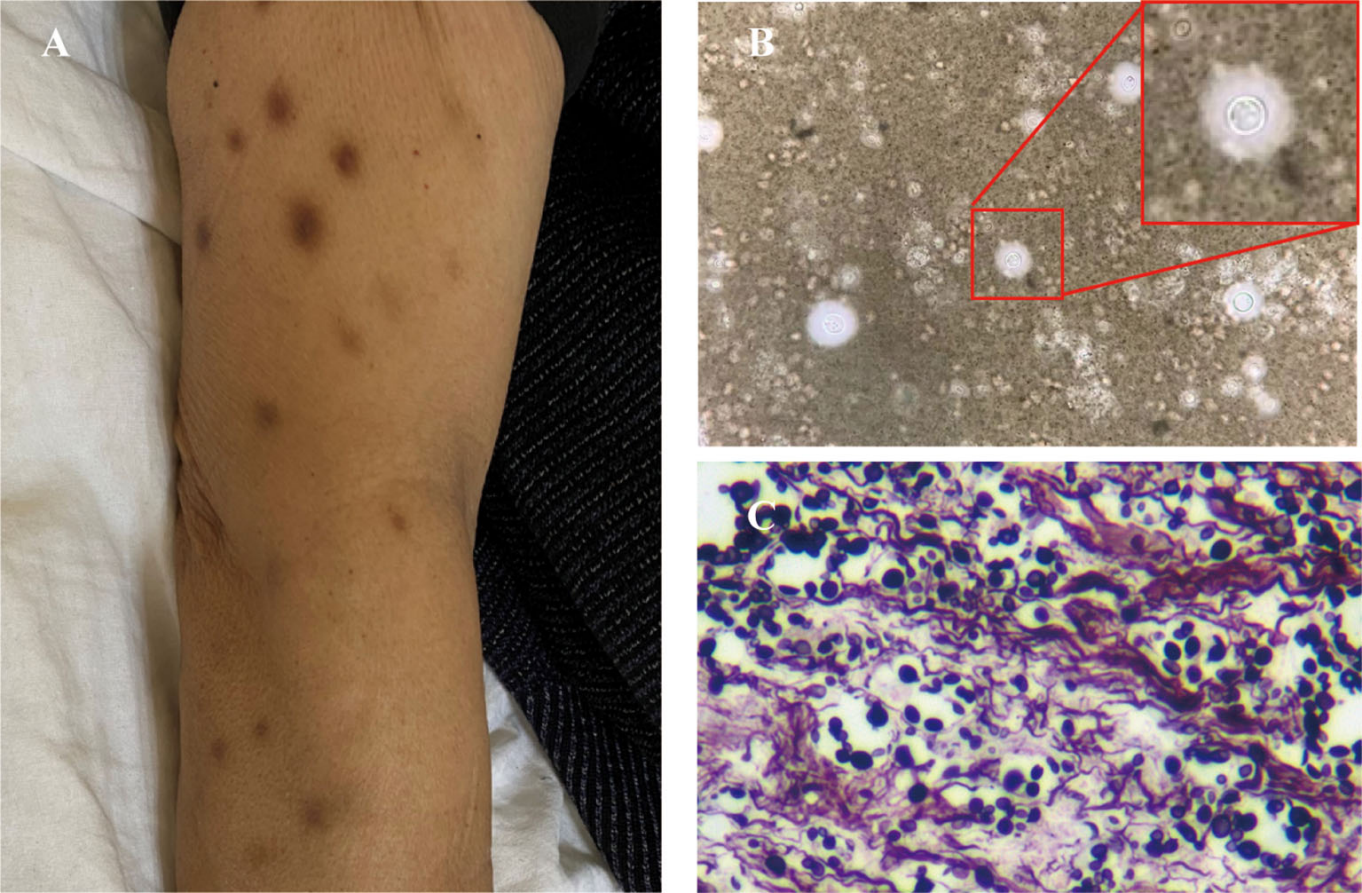
Clinical and histopathological findings in Case 1. **(A)** Multiple 0.5-1.5 cm brownish subcutaneous nodules distributed on the upper extremities. **(B)** India ink staining of abscess aspirate demonstrates encapsulated yeast forms (outline) with characteristic prominent halos (original magnification ×100). **(C)** GMS staining of skin biopsy confirms numerous oval yeast cells with typical morphology of *Cryptococcus* species (original magnification ×20).

A 52-year-old man presented with a one-month history of persistent fever (peak 39 °C), back pain, and multiple disseminated brown cutaneous nodules. Four years prior, he underwent thymectomy for thymoma and subsequently developed MG, requiring long-term tacrolimus. One year ago, he received radioactive seed implantation for pleural recurrence, followed by pulmonary radiofrequency ablation for a metastatic nodule two months before admission. His comorbidities included hypertension, diabetes, and coronary artery disease. Physical examination revealed multiple 0.5-1.5 cm non-tender, brownish subcutaneous nodules distributed over the face, trunk, and extremities, some demonstrating fluctuance or necrosis (**Figure 1A**). A tender, fluctuant 8 cm mass was palpable on the left lateral chest wall. Neurological examination showed tenderness over the T4 vertebra and right paravertebral region, with bilateral lower extremity muscle strength graded as 4/5.

**Figure 2:**
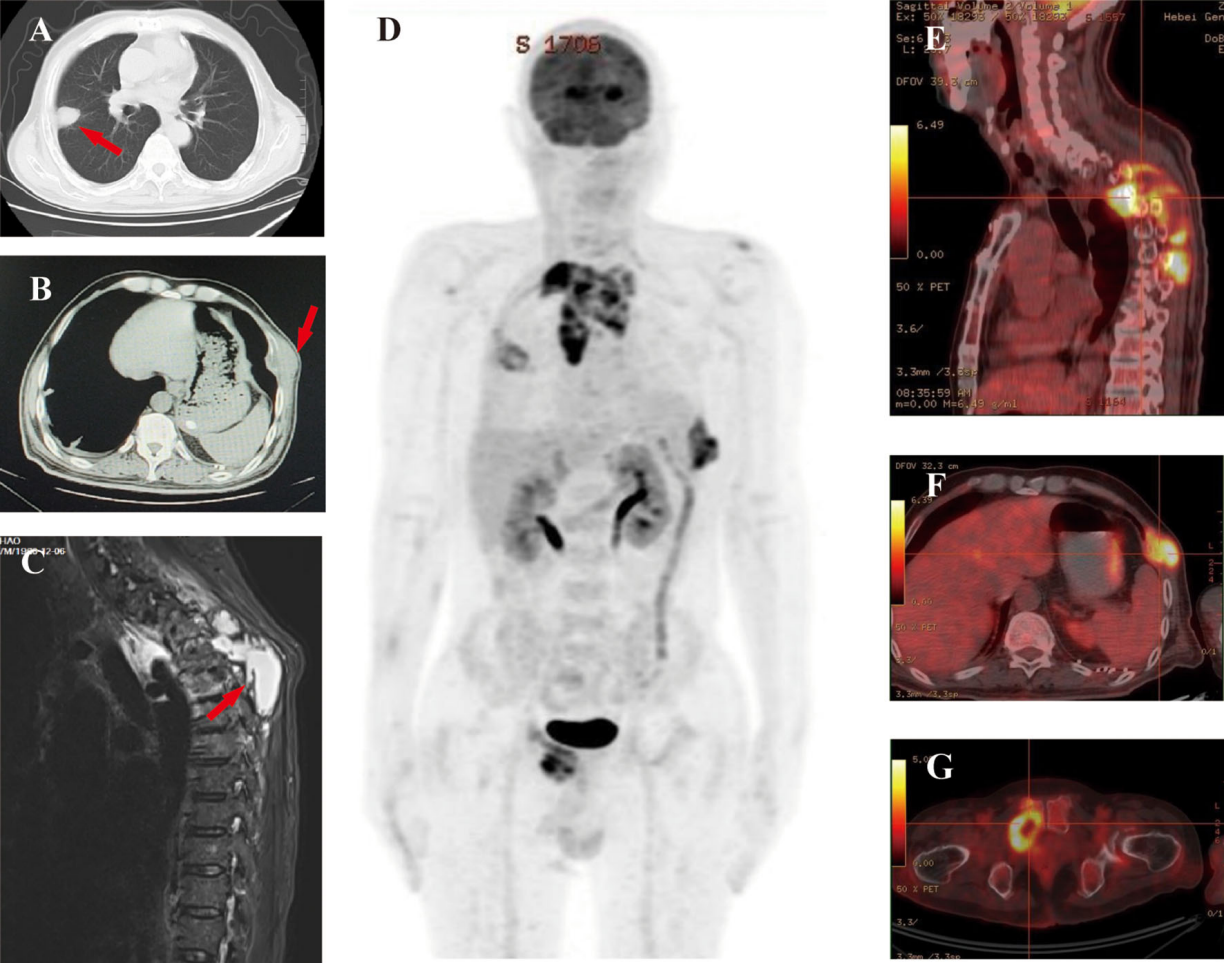
Radiological imaging of Case 1. **(A)** Chest CT demonstrates a 1.2 cm nodule (arrow) in the right upper lobe with surrounding ground-glass opacity. **(B)** Chest CT reveals localized left pleural thickening and an 8 cm chest wall mass (arrow). **(C)** Sagittal T2-weighted MRI of the thoracic spine shows inflammatory changes involving T3-T5 vertebral bodies and surrounding tissues with pathological compression fracture at T4 (arrow). **(D)** PET-CT further identified widespread hypermetabolic lesions, with SUVmax of 10.6 in the upper thoracic region **(E)**, 8.2 in bilateral chest walls **(F)**, and 9.5 in the right pubic ramus **(G)**.

Laboratory investigations (**Table 1**) revealed a CD4+ T cell count of 181 cells/μL. Serum CrAg testing was positive, while 1,3-β-D-glucan, galactomannan, and *Mycobacterium tuberculosis* T-SPOT.TB tests returned negative results. Chest computed tomography (CT) revealed post-thymectomy changes and a right upper lobe nodule (**Figure 2A**) with an adjacent left chest wall mass (**Figure 2B**). Thoracic spine magnetic resonance imaging (MRI) revealed inflammatory involvement of the vertebral bodies (T3–T5) and paravertebral soft tissues (T2–T8), including a pathological T4 compression fracture (**Figure 2C**). Positron emission tomography (PET)-CT further identified widespread hypermetabolic lesions, with maximum standardized uptake values (SUVmax) of 10.6 in the upper thoracic region, 3.7 in the left acromion, 8.2 in bilateral chest walls, and 9.5 in the right pubic ramus, along with multiple subcutaneous and intramuscular foci (SUVmax 2.3-3.1) in the right forearm and bilateral lower extremities (**Figures 2D-G**).

**Table 1:** Admission laboratory profiles of three myasthenia gravis patients with cryptococcosis in our case series.

Parameters	Case 1	Case 2	Case 3	Reference range
Blood routine test				
Leukocytes (× 10^9^ /L)	5.10	5.64	7.79	3.5-9.5 × 10^9^/L
Neutrophils (× 10^9^ /L)	4.09	3.37	4.61	1.8-6.3 × 10^9^/L
Lymphocytes (× 10^9^ /L)	0.69	1.68	2.54	1.1-3.2 × 10^9^/L
Hemoglobin (g/L)	90.6	97.3	106.0	115–150 g/L
Platelets (× 10^9^ /L)	363	354	179	125-350 × 10^9^/L
Lymphocyte subset counts				
CD4+ T cells (cells/μL)	181	604	1200	550-1440 (cells/μL)
CD8+T cells (cells/μL)	141	762	497	320-1250 (cells/μL)
Biochemical indexes				
Albumin (g/L)	33.3	39.5	42.7	40–55 g/L
Globulin(g/L)	31.1	26.3	20.5	20–40 g/L
Alanine Aminotransferase (U/L)	21	11	11	9–50 U/L
Creatinine (μmol/L)	58.9	31.2	98	41-73 μmol/L
Glucose (mmol/L)	5.76	5.24	5.05	3.9-6.1 mmol/L
Inflammatory markers				
Erythrocyte Sedimentation Rate (mm/h)	100	36	10	0–20 mm/h
C-Reactive Protein (mg/L)	63	17.46	<0.5	≤ 6 mg/L
Serum Amyloid A (mg/L)	> 300	92.37	ND	0–10 mg/L
CrAg	1: 80	1: 40	1: 40	
Cerebrospinal fluid analysis				
CSF Pressure (mmH_2_O)	140	120	ND	80-180mmH_2_O
White Blood Cell (× 10^6^/L)	48	4	ND	0-8 × 10^6^/L
Mononuclear Cells (%)	38	3	ND	60-100%
Polymorphonuclear Leukocytes (%)	10	1	ND	0-10%
Lactate Dehydrogenase (U/L)	44.11	25.16	ND	< 40 U/L
Glucose (mmol/L)	2.41	2.82	ND	2.2-3.9 mmol/L
Protein (g/L)	1.35	0.21	ND	0.12-0.6 g/L
Adenosine Deaminase (U/L)	2.2	0.5	ND	< 8 U/L
Lactate (mmol/L)	2.51	1.23	ND	0.7-2.1 mmol/L

ND, not done.

Microscopic examination of pus from the cutaneous abscesses (left chest wall and back) revealed encapsulated yeast on India ink staining (**Figure 1B**). Metagenomic next-generation sequencing (mNGS) identified *C. neoformans* with sequence counts of 30 and 65 in above pus specimens, respectively. Fungal culture and drug susceptibility testing confirmed *C. neoformans* susceptible to fluconazole, voriconazole, flucytosine, and amphotericin B. Skin biopsy demonstrated characteristic yeast forms positive for Grocott's methenamine silver (GMS) staining (**Figure 1C**). Lumbar puncture showed inflammatory CSF changes consistent with CNS involvement despite negative cultures (**Table 1**). These findings established a definitive diagnosis of disseminated cryptococcosis. WES of peripheral blood demonstrated a missense point mutation of c.C56T in exon 2 of the FAS (NM_000043.6) gene, resulting in mutation of amino acid of protein 19 encoded by the gene from serine to leucine (p. S19L).

**Figure 3:**
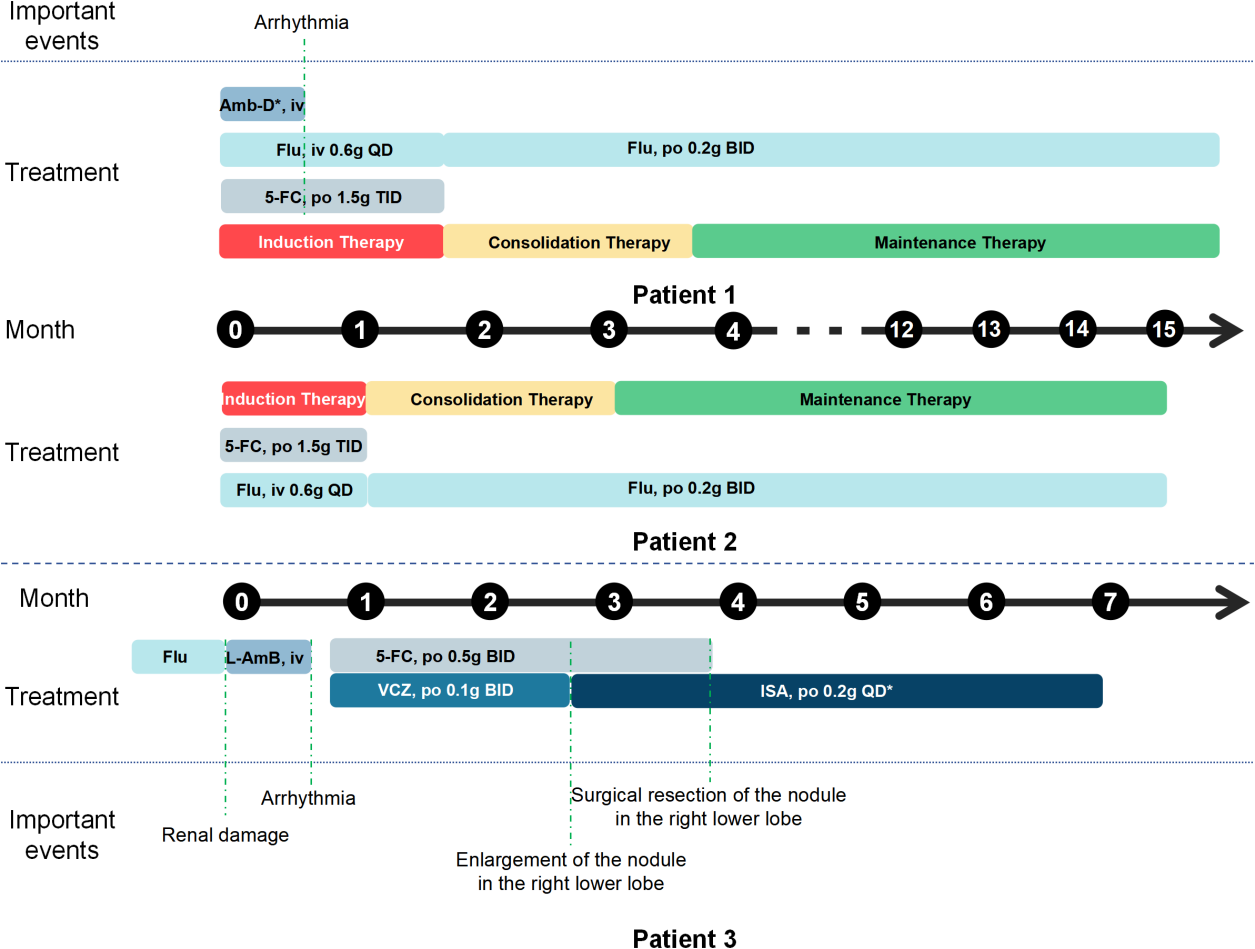
Treatment timeline for three patients. Patients 1 and 2 had disseminated cryptococcosis and were treated with regimens including induction, consolidation, and maintenance therapy. Amb-D*: Amphotericin B deoxycholate. Patient 1 received an initial dose of 5 mg QD, which was gradually increased to 25 mg QD. ISA*: Isavuconazole. Administered at 200 mg Q8h for the first two days, then switched to 200 mg QD.

The patient received initial triple antifungal therapy with fluconazole (600mg daily), flucytosine (1.5g three times daily), and amphotericin B (25mg daily). After 12 days, amphotericin B was discontinued due to drug-induced arrhythmia, and treatment continued with fluconazole plus flucytosine for four weeks until CSF normalization, followed by maintenance fluconazole (200mg twice daily). Therapeutic drug monitoring (TDM) revealed initial elevated tacrolimus levels (20.8μg/L), which was subsequently managed through careful dose titration to maintain therapeutic levels between 5-10μg/L. After 14 months of therapy (including a 6-week induction phase, an 8-week consolidation phase, and a 42-week maintenance phase), the patient achieved complete clinical resolution. The treatment flowchart is shown in **Figure 3**.

#### 3.1.2 Case 2

A 55-year-old woman presented with a one-month history of unexplained right costal pain, without fever, cough, or sputum production. She had a medical history of thymoma treated with thymectomy nine months prior, followed by a diagnosis of MG. She was on azathioprine and methylprednisolone immunosuppression treatment. Initial evaluation at an outside hospital two weeks prior had revealed fibrous and inflammatory granulation tissue on pathological examination of a right rib lesion. Concurrent pathological analysis of a left pulmonary nodule identified *Cryptococcus* species, with positive periodic acid-Schiff and GMS staining. Due to inadequate clinical response to fluconazole monotherapy, she was subsequently transferred to our hospital for further management. Laboratory parameters showed positive serum CrAg, and others were summarized in **Table 1**. PET-CT demonstrated multiple hypermetabolic pulmonary nodules, diffuse osteolytic lesions with associated metabolic activity, and focal spinal hypermetabolism. Notably, lumbar biopsy and CSF analysis excluded CNS involvement (**Table 1**), and no pathogenic or likely pathogenic variants were identified. The patient responded well to combination antifungal therapy with fluconazole (600mg daily) and flucytosine (1.5g three times daily), showing significant symptomatic improvement. She was discharged on consolidation and maintenance therapy using fluconazole (200mg twice daily) with continued outpatient monitoring for 1 year (**Figure 3**).

#### 3.1.3 Case 3

**Figure 4:**
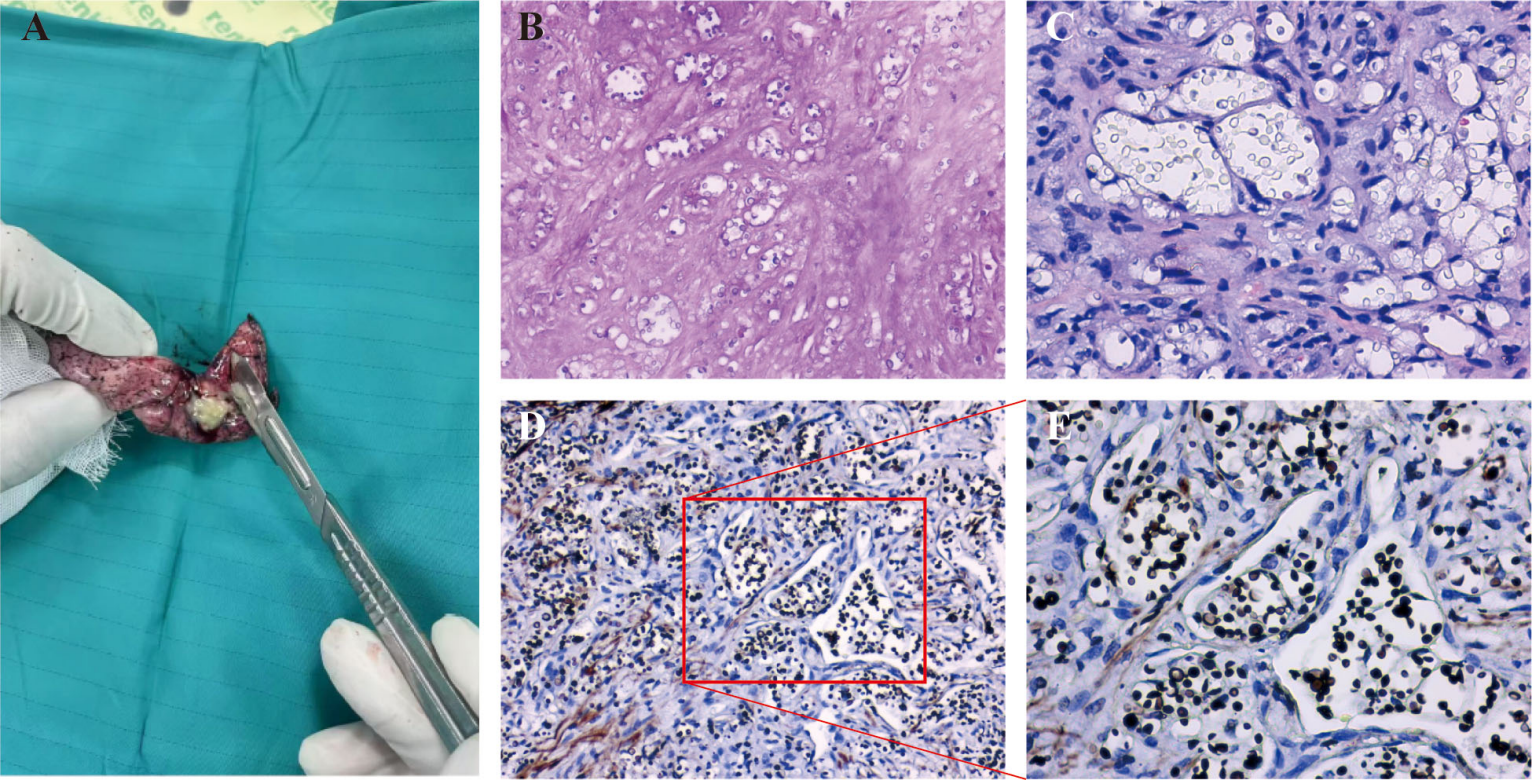
Intraoperative and histopathological findings of pulmonary cryptococcosis in Case 3. **(A)** Resected pulmonary nodule. **(B, C)** Hematoxylin and eosin staining reveals granulomatous inflammation with scattered round, translucent yeast-like structures, consistent with *Cryptococcus* species **(B)** original magnification ×20; **(C)** original magnification ×40). **(D, E)** GMS staining confirms numerous oval, black-staining fungal organisms (outline) with characteristic morphology of *Cryptococcus* **(D)** original magnification ×20; **(E)** original magnification ×40).

A 63-year-old woman with MG, diagnosed seven months earlier and maintained on prednisone and tacrolimus therapy, developed pulmonary cryptococcosis confirmed by biopsy of a right lower lobe nodule and positive serum CrAg four months after her MG diagnosis. She was intolerant to initial treatment of fluconazole and liposomal amphotericin B (L-Amb) due to drug-induced nephrotoxicity. Therefore, the alternative treatment involves the use of voriconazole (100mg twice daily; trough levels maintained at 1–2 mg/L) or isavuconazole (200mg every eight hours for two days, followed by 200mg daily) combined with flucytosine (500mg twice daily). However, follow-up CT imaging demonstrated non-resolution of the right lung nodules and development of new bilateral nodules. Ultimately, the patient underwent surgical resection of the residual pulmonary nodules (**Figure 4A**), with histopathological examination confirming cryptococcal granulomas (**Figures 4B-E**), followed by a three-month course of isavuconazole (200mg daily) therapy. The key therapeutic intervention for this patient is shown in **Figure 3**.

### 3.2 Literature review

A systematic literature review identified 15 relevant articles, one of which was excluded due to inaccessibility of the full text and abstract, leaving 14 cases for analysis (Jia et al., 2023; Huong et al., 2017; Shah et al., 2015; Lorenzoni et al., 2011; Takenaka et al., 2010; Yip et al., 1998; Schmidt and Padberg, 1998; Rowland et al., 1965; Narayanan et al., 2011; Ieronymaki et al., 2021; Youssef et al., 2024; Soni et al., 2020; Asif et al., 2019; Drogari-Apiranthitou et al, 2024). Combined with our case series of three patients, a total of 17 cases were included in the final analysis.

**Table 2** summarizes the patient demographics, associated immune disorders, MG therapies, sites of involvement, diagnostic methods, and treatment details. The median age of the patients was 57.1 years (range: 34-79), without a sex predominance (male 52.9%, 9/17). All patients were HIV-negative, and 41.2% (7/17) had diabetes mellitus. The most common manifestation was CNS involvement (52.9%), followed by pulmonary (47.1%) and cutaneous (23.5%) disease. Among the nine patients with CNS involvement, CSF cultures were positive in 66.7% (6/9). CrAg testing was performed in 15 patients, demonstrating positivity in all 12 serum samples and all six CSF samples tested, with three cases showing concurrent positivity in both serum and CSF. The remaining two cases were diagnosed *via* bronchoalveolar lavage and pleural biopsy histopathology, respectively. Isolated prosthetic joint, pleural, and prostatic involvement were each observed in one case. Disseminated infection occurred in six patients (35.3%), all of whom had CSF involvement. Six MG patients had thymus-related comorbidities, including five with thymoma and one with thymic carcinoma. Corticosteroids were administered to 76.5% (13/17) of MG patients, consisting of prednisone in 11 cases and methylprednisolone in two cases. Azathioprine was concurrently used in 47.1% (8/17) of patients, while one patient received additional eculizumab therapy. One patient required triple immunosuppressive therapy (prednisone, azathioprine, and eculizumab). Among patients with CNS involvement or disseminated infection, the most frequently employed initial regimen was amphotericin B combined with fluconazole/flucytosine dual therapy, accounting for 4/10 cases (40%). In contrast, for those with single-site infection, amphotericin B monotherapy predominated as the initial approach in 4/7 patients (57.1%). Fluconazole monotherapy was uniformly the most common subsequent treatment. Surgical intervention was performed in three patients, specifically for prostate abscess drainage, debridement of hip prosthesis infection, and debridement of cutaneous ulcers. The majority of patients achieved favorable clinical outcomes. However, one patient with concurrent CNS involvement and bloodstream infection experienced clinical deterioration despite combination therapy with amphotericin B and fluconazole. Subsequent susceptibility testing revealed fluconazole-resistant *Cryptococcus neoformans*, which likely constituted a primary contributing factor to therapeutic failure.

**Table 2:** Overview of case series and literature review of myasthenia gravis with cryptococcosis.

Author (Year)	Age/Sex	Comorbidities	Immunosuppressive therapy	Infection sites	CD4+ T cell (cells/μL)	CrAg test	Microbiology for cryptococcus			Induction /Initial therapy	Maintenance therapy	Prognosis	
							Molecular test	Histology	Culture				
Rowland et al (1965)	48/F	MG, Thym	CRT	CNS	NM	Blood (+)	NM	NM	CSF (+)	L-AmB	NM	Expired for bacterial pneumonia
Yip et al (1998)	71/M	MG, DM	PRED, AZA	Prostate, CNS	NM	Blood (+), CSF (+)	NM	Prostate Abscess (+)	Blood (+), CSF (+)	AmB, Flu	Flu	Improved
Schmidt et al (1998)	36/M	MG, ThyCa	PRED, AZA	CNS	20	CSF (+)	NM	NM	NM	Flu	NM	Improved
Takenaka et al (2010)	59/Fe	MG, Thym	PRED	Lung	NM	Blood (+)	NM	NM	NM	ITC	NM	Improved
Lorenzoni et al (2011)	42/F	MG	PRED, AZA	CNS	342	Blood (+), CSF (+)	NM	CSF (+)	CSF (+)	AmB	NM	Improved
Narayanan et al. (2011)	79/F	MG	PRED, MMF	CNS, Lung, Multifocal skin lesions	NM	CSF (+)	NM	BALF (+)	BALF (+), Blood (+), CSF (+)	L-AmB	Flu	Improved
Drogari-Apiranthitou et al (2024)	61/M	MG	PRED	Left middle finger	NM	Blood (+)	PCR (+)	Skin lesions (+)	Skin lesion (+), Blood (-)	Flu	NM	Improved
Shah et al (2015)	77/F	MG, DM	PRED, AZA	Hip prosthesis	NM	Blood (+)	NM	NM	Hematoma (+), Joint capsule (+)	L-AmB	Flu	Improved
Huong et al (2017)	39/M	MG, DM, HCV, TB	MP, AZA	CNS, Multifocal skin lesions	236	CSF (+)	NM	Skin lesions (+)	Skin lesions (+)	AmB, Flu	Flu	Improved
Asif et al (2019)	71/M	MG, DM	PRED, MPA	Lung	NM	Blood (+)	NM	BALF (+)	NM	L-AmB	Flu	Improved
Soni et al (2020)	61/M	MG, DM	LDS, AZA	Lung	NM	NM	NM	BALF (+)	NM	L-AmB	Flu	Improved
Ieronymaki et al (2021)	74/F	MG, DM, AB	PRED, MMF	CNS, Lung	NM	Blood (+), CSF (+)	NM	NM	Blood (+), CSF (+)	L-AmB, Flu	NM	Worsened
Jia et al (2023)	49/M	MG, Thym	PRED, TAC	Pleura	197.25	NM	mNGS (+)	Pleura (+)	Pleural Effusion (-)	AmB	Flu	Improved
Youssef et al (2024)	34/M	MG	PRED, AZA, ECU	CNS	483	Blood (+)	PCR (+)	NM	CSF (+)	L-AmB, 5-FC	Flu	Improved
Our Case 1	52/M	MG, DM, Thym	TAC	CNS, Lung, Multifocal skin lesions, Spine	181	Blood (+)	mNGS (+)	Skin lesions (+)	Abscesses (+), Blood (-)	Flu, 5-FC	Flu	Improved
Our Case 2	55/F	MG, Thym	MP, AZA	Lung, rib	603	Blood (+)	NM	PN	CSF (-)	Flu, 5-FC	Flu	Improved
Our Case 3	63/F	MG	MP, TAC	Lung	1200	Blood (+)	NM	PN	NM	VRC, 5-FC	ISA	Improved

LDS: AmB, Amphotericin B; AZA, Azathioprine; BALF, Bronchoalveolar lavage fluid; CNS, Central nervous system; CSF, Cerebrospinal fluid; CrAg, Cryptococcal antigen; CRT: Chemoradiation; DM: Diabetes mellitus; ECU: Eculizumab; Flu, Fluconazole; 5-FC, Flucytosine; HCV, Hepatitis C; L-AmB, Liposomal amphotericin B; ISA, Isavuconazole; ITC, Itraconazole; LDS, Low dose steroids; MG, Myasthenia gravis; MMF, Mycophenolate mofetil; mNGS, Metagenomic next-generation sequencing; MP, Methylprednisolone; MPA, Mycophenolic acid; NM, Not mentioned; PCR, Polymerase chain reaction; PN, Pulmonary nodule; PRED, Prednisone; TAC, Tacrolimus; TB, Tuberculosis; ThyCa, Thymic carcinoma; Thym, Thymoma; VRC, Voriconazole

## 4 Discussion

Cryptococcosis poses a significant threat to immunocompromised individuals, particularly MG patients undergoing immunosuppressive therapy. Our study represents the first comprehensive literature review summarizing cases of MG complicated with cryptococcal infection. Our analysis of three new cases combined with 14 published literature reports (Jia et al., 2023; Huong et al., 2017; Shah et al., 2015; Lorenzoni et al., 2011; Takenaka et al., 2010; Yip et al., 1998; Schmidt and Padberg, 1998; Rowland et al., 1965; Narayanan et al., 2011; Ieronymaki et al., 2021; Youssef et al., 2024; Soni et al., 2020; Asif et al., 2019; Drogari-Apiranthitou et al., 2024) reveals that affected patients are typically middle-aged (50–60 years) with no gender predominance. The infection most commonly involves the CNS, followed by pulmonary and cutaneous manifestations, with approximately one-third of cases progressing to disseminated disease that invariably involves the CSF, underscoring the aggressive nature of cryptococcosis in this setting. Notably, the overwhelming majority of affected patients were receiving dual or triple immunosuppressive regimens, highlighting the delicate balance between managing MG and preventing opportunistic infections. These findings emphasize the need for heightened clinical vigilance, prompt diagnostic evaluation, and individualized treatment strategies in this vulnerable population.

The development of cryptococcosis in MG patients is multifactorial, involving both iatrogenic immunosuppression and underlying immune dysregulation. Immunosuppressive agents such as glucocorticoids, azathioprine, and tacrolimus, cornerstones of MG management, significantly impair host defenses against fungal infections. Thymectomy-associated MG, as observed in three patients of our study, represents a well-documented but incompletely understood phenomenon with reported incidence rates of 1.0%-9.1% (Kim et al., 2021), may further exacerbate immune dysfunction, though the precise mechanisms remain unclear. Potential mechanisms include postoperative immune dysregulation, persistence of autoreactive T-cell clones, or undetected subclinical autoimmune status prior to surgery (Kim et al., 2021; Mineo et al., 2018).

CD4+ T lymphocytes play a pivotal role in anti-cryptococcal immunity, primarily through their differentiation into Th1 and Th17 effector subsets. These subsets secrete critical cytokines, including interleukin-2, tumor necrosis factor-α, and interferon-gammar (IFN-γ), which orchestrate macrophage phagocytic activity and directly suppress fungal proliferation (Rohatgi and Pirofski, 2015). In our study, the gradient of disease severity of patients is correlated with CD4+ T cell depletion, mirroring findings in HIV-associated cryptococcosis. Based on a comparative analysis of these 17 clinical cases, patients with disseminated cryptococcosis exhibited significantly lower CD4+ T cell counts than those with localized cryptococcal infections. This aligns with existing evidence suggesting that CD4+ T cell counts below 400 cells/μL substantially increase the risk of disseminated cryptococcosis in non-HIV immunocompromised hosts (Denham and Brown, 2018; Wang et al., 2021).

WES analysis of our Case 1 identified a heterozygous FAS mutation (p.S19L), which plays a central role in the physiological regulation of programmed cell death. This variant linked to autoimmune lymphoproliferative syndrome (Paskiewicz et al., 2023). Such patients fail to adequately regulate immune cell populations, frequently developing aberrant lymphocyte proliferation. While potentially contributing to immune dysregulation, its specific role in cryptococcal susceptibility requires further investigation. This finding underscores our incomplete understanding of genetic-immune interactions in cryptococcosis development among MG patients. The host's defense against *Cryptococcus* depends critically on IFN-γ-mediated Th1 immunity, where elevated CSF IFN-γ levels correlate with better treatment outcomes in cryptococcal meningitis (Rohatgi and Pirofski, 2015; Okurut et al., 2025). Our case series showed negative anti-IFN-γ autoantibodies, which are recognized to disrupt macrophage activity and intracellular microbial killing, as demonstrated in *Non-tuberculous mycobacteria* and *T. marneffei* infections (Tang et al., 2010; Kampitak et al., 2011). More recently, anti-granulocyte-macrophage colony-stimulating factor autoantibodies have emerged as significant predictors of both cryptococcal susceptibility and poorer survival outcomes, independent of *Cryptococcus* species (Jiang et al., 2024; Arango-Franco et al., 2024).

Cryptococcosis in MG patients often mimic malignancies (e.g. lymphoma, metastatic malignancies) and other infections (e.g. tuberculosis) (Joo et al., 2015; Zhang et al., 2012). In our series, hypermetabolic lesions on PET-CT initially raised suspicion for metastatic thymoma or other malignancies, delaying appropriate treatment. CrAg testing proved invaluable, with 88.2% positivity among 17 MG patients. It is demonstrated that particular utility of mNGS in extrapulmonary cases (93.5% sensitivity and 96.0% specificity for CNS cryptococcosis), offering rapid species identification and aiding in the diagnosis of culture-negative infections (Gan et al., 2022). Histopathology and fungal culture remain the diagnostic gold standards, but their clinical utility is limited by the invasive nature of tissue sampling and prolonged culture duration. For example, in our Case 3, persistent pulmonary nodules despite antifungal therapy necessitated surgical resection to exclude malignancy, highlighting the diagnostic ambiguity that often accompanies cryptococcosis in immunocompromised hosts.

Infection is the most significant trigger for worsening MG (Gilhus et al., 2018). Additionally, certain antibiotics can impair neuromuscular transmission, exacerbating muscle weakness in MG patients (Gilhus et al., 2018). Managing cryptococcosis in these individuals also requires careful balancing of antifungal efficacy against the potential toxicity of immunosuppressants. Induction therapy utilizing L-Amb (with amphotericin B lipid complex or amphotericin B as alternative) and flucytosine, as recommended by current guidelines, was employed in CNS and disseminated cryptococcal infection (Chang et al., 2024). However, our Case 1 developed arrhythmias secondary to amphotericin B, necessitating a switch to fluconazole and flucytosine. TDM was critical in this patient due to fluconazole-mediated inhibition of tacrolimus metabolism, which led to supratherapeutic tacrolimus levels and potential toxicity. In our Case 3, the patient developed renal toxicity from liposomal amphotericin B and fluconazole, displayed unresponsive to other azole antifungal medications, and ultimately required surgical resection, highlighting the importance of individualized treatment strategies and close monitoring in such cases. The optimal duration of antifungal therapy remains uncertain, particularly in immunocompromised hosts. While undetectable CrAg levels may serve as a pragmatic endpoint, this approach requires validation in larger studies.

Given the limited published cases of MG with cryptococcosis, current case reports demonstrate substantial heterogeneity in both treatment regimens and the application of immunosuppressive agents. Analysis of the included patients indicates that initial treatment most commonly utilized amphotericin B-based regimens, either as monotherapy or in combination. The majority of patients achieved favorable outcomes. If localized lesions develop, a comprehensive assessment of the necessity for surgical intervention must be conducted. In such patients, immunosuppressant use serves a dual role: it constitutes one of the risk factors for cryptococcal invasion due to compromised immunity, while simultaneously functioning as a protective measure against pathological inflammatory responses during treatment (Hargarten et al., 2023). Overly rapid discontinuation of immunosuppressants may precipitate cryptococcal inflammatory syndromes. Among the included cases, only one patient developed immune reconstitution syndrome following prednisone cessation. This phenomenon may be attributed to the sustained suppression of T-cell activation and proinflammatory cytokine release by immunosuppressive therapy, thereby preventing the occurrence of excessive inflammation.

In conclusion, cryptococcosis in MG patients presents complex diagnostic and therapeutic challenges due to immune dysfunction and immunosuppressive therapy. Achieving optimal therapeutic balance between treatment of MG and cryptococcosis remains paramount in each individual case. A multidisciplinary approach combining rapid diagnostics, TDM-guided treatment adjustment, and personalized antifungal regimens is crucial. Further research is needed to optimize risk stratification and management strategies for this vulnerable population.

## Data Availability

The raw data supporting the conclusions of this article will be made available by the authors, without undue reservation.
